# Attentional Processing of Food Cues in Overweight and Obese Individuals

**DOI:** 10.1007/s13679-012-0011-1

**Published:** 2012-03-28

**Authors:** Ilse M. T. Nijs, Ingmar H. A. Franken

**Affiliations:** 1 Institute of Psychology, Erasmus University Rotterdam, P.O. Box 1738, 3000 DR Rotterdam, the Netherlands; 2 Child and Adolescent Psychiatry/Psychology (KP-2-881), Erasmus Medical Center—Sophia Children’s Hospital, P.O. Box 2060, 3000 CB Rotterdam, the Netherlands

**Keywords:** Overweight, Obesity, Overeating, Food, Attention, Craving, Hunger, Satiety, Information processing, Binge eating, Addiction, Incentive sensitization, Cue reactivity, Event-related potentials, Stroop task, Visual probe task, Eye tracking, P300, Food intake

## Abstract

The incentive sensitization model of obesity hypothesizes that obese individuals in the western world have acquired an enhanced attention bias to food cues, because of the overwhelming exposure to food. This article gives an overview of recent studies regarding attention to food and obesity. In general, an interesting approach-avoidance pattern in food-related attention has been found in overweight/obese individuals in a number of studies. However, it should be noted that study results are contradictory. This might be due to methodological issues, such as the choice of attention measurements, possibly tapping different underlying components of information processing. Although attention research is challenging, researchers are encouraged to further explore important issues, such as the exact circumstances in which obese persons demonstrate enhanced attention to food, the directional relationship between food-related attention bias, overeating and weight gain, and the underlying involvement of the reward system. Knowledge on these issues could help improve treatment programs.

## Introduction

The prevalence of obesity has more than doubled since the 1980s worldwide [1]. The fundamental cause of obesity is an imbalance in energy intake and energy expenditure. As energy expenditure does not seem to have changed substantially over the past 30 years, an increased energy intake is believed to be the main factor explaining the current obesity epidemic [2]. It is generally assumed that energy intake has changed because of changes in the environment: compared to the 1980s, western(ized) environments are now characterized by an abundant availability of highly rewarding, generally high-fat/high-sugar foods, which are advertised aggressively. That is, everyone in the western world is now exposed continuously and heavily to food-related cues.

All humans are naturally attracted to rewarding foods, at least to some extent. Food responsiveness is regulated in a brain network, consisting of mesolimbic and frontal brain regions, which is referred to as the reward system. Communication within the brain reward network happens through neurotransmitters such as dopamine, which is thought to be primarily involved in appetitive (“wanting”) responses to rewarding stimuli [3•].

It is well known that the reward system is implicated in drug addiction. Several addiction theories acknowledge that a drug-related cue reactivity, and particularly the subjective experience of craving, is a central feature of addiction, involved in the inability of addicted persons to stop the drug use and in relapse [4]. One of these theories is the incentive sensitization theory [5, 6]. In short, according to this theory the repeated exposure to drugs leads to a process of incentive sensitization in the dopamine-driven brain reward system. Through a process of associative conditioning, not only drugs themselves, but all stimuli associated with drug use acquire incentive salience qualities. That is, the mere exposure to drug-related stimuli elicits the release of dopamine in the brain reward system, and because of this these stimuli become attention-grabbing, induce subjective craving, and ultimately elicit approach behavior. According to this theory, an attention bias to drug-related cues is, as much as subjective craving, a central feature of addiction, and is also directly related to dopamine activity in the reward system. A wealth of research shows that addicted persons (to all kinds of drugs) are generally characterized by an attention bias for relevant drug-related stimuli, which is associated with subjective craving and predicts relapse [7, 8•].

There are similarities between addictive behavior and overeating and obesity. As addicts, persons with an overeating problem report cravings for, generally high-calorie, food and loss of control over eating. For many people, it is difficult to resist palatable, high-calorie foods, although the negative consequences of indulging in these foods are well known. Relapse rates are equally high in weight loss programs as in addiction treatment programs [9, 10, 11•]. On a neurobiological level, several studies point to similar deficits in dopamine-based reward system functioning in persons with an addiction and an overeating problem/obesity [12, 13]. According to some obesity researchers, neurocognitive addiction models, such as the incentive sensitization theory, might be applicable to obesity [3•]. This means that, similar to addiction, an attention bias to rewarding foods might play an important role in the development and maintenance of overeating behavior and weight gain/obesity. During the last few years the relationship between attention bias to food-related stimuli and obesity has received increasing attention. This review gives a critical overview of studies concerning the attentional processing of food cues in obese and overweight individuals over the past 3 years (2009–2011). The general hypothesis of these studies is that overweight/obese individuals demonstrate an enhanced attention bias to highly rewarding, generally high-calorie foods, and that this attention bias is associated with subjective ratings of food craving.

## Attention Bias Studies in Obese Versus Normal Weight Individuals

Table 1 gives an overview of studies that have directly compared, otherwise healthy, overweight/obese and normal weight persons with regard to food-related attention bias. Various tasks and techniques were used to assess two different components of attention to food-related stimuli: the initial orientation of attention (ie, the immediate and automatic detection of food-related stimuli) and the maintenance of attention (ie, the difficulty to disengage attention from food-related stimuli). Interestingly, several studies found evidence for an enhanced oriented attention to, particularly high-calorie, food stimuli in overweight/obese persons. This was found by Castellanos et al. [14] and Werthmann et al. [15], using an eye-tracking procedure, by Nijs et al. [16•], using a visual probe task, and by Nijs et al. [17] using the amplitude of the P200 event-related potential (ERP; see further for an explanation of attention methodologies). Also interesting is that in some studies evidence was found for a reduced maintenance of attention, that is, an avoidance of or a shift away from high-calorie food-related stimuli in overweight/obese persons. This was reported by Nijs et al. [16•], using the amplitude of the P300 ERP, and by Werthmann et al. [15], who investigated the duration of initial eye fixations to food-related stimuli. An approach-avoidance tendency or a motivational ambivalence toward high-calorie food stimuli in overweight/obese persons makes sense, as these persons might experience a conflict between the desire to eat (as reflected in a strong initial orientation toward food) and the desire to lose weight (as reflected in a subsequent shift away from food). A motivational ambivalence is also seen, for instance, in unsuccessful dieters [18], self-reported chocolate cravers [19], and alcoholics who try to abstain from alcohol [20], suggesting that it might be a typical response to desired but “forbidden” substances.

**Table 1:** A summary of food-related attention bias studies in OB/OW visual probe task individuals between 2009–2011

Study	Study groups	Measure of attention	Stimuli	Attention parameters (oriented/maintained attention)	Hunger/satiety intervention	Main findings: difference between OW/OB and NW subjects	Main findings: positive association between attention bias and subjective craving
Castellanos et al. [14]	18 OB and 18 NW women	ET during performance of a VPT	Pairs of pictures of HC/LC foods and non-food items	ET direction bias	Hunger (> 8 h) + satiety (shake 400 kcal; within-subjects design)	ET direction + duration bias:	Direction/duration bias ~ hunger
				ET duration bias		Hunger: OB = NW	
				VPT RT bias 2,000 ms		Satiety: reduced in NW; retained in OB	
Werthmann et al. [15]	22 OW/OB and 29 NW women	ET during performance of a VPT	Pairs of pictures of high-fat foods and non-food items	ET direction bias	Restrain from eating 2 h prior to testing; satiated with a lunch (400–500 kcal) before testing	ET direction bias: OW/OB > NW	In OW/OB: ET duration bias ~ craving
				ET first fixation duration bias		ET first fixation duration bias: OW/OB < NW	
				ET duration bias			
				VPT RT duration bias 2,000 ms			
Nijs et al. [16•]	26 OW/OB and 40 NW women	ET, VPT, ERP	Pairs of pictures of HC foods and non-food items	ET direction bias	Hunger (17 h) + satiety (shake 600 kcal; between-subjects design)	RT bias 100 ms: OW/OB > NW, especially in hunger	In NW: P300 bias ~ hunger
				ET duration bias		P300 bias:	In OW/OB: VPT direction bias ~ hunger
				VPT RT direction bias 100 ms		Hunger: OW/OB < NW	
				VPT RT duration bias 500 ms			
				P300 ERP bias		Satiety: OW/OB > NW	
Nijs et al. [17]	20 OB and 20 NW men and women	ERP during a food-modified Stroop task	Words, referring to HC foods and non-food items	P200 ERP	Light meal 2 h prior to testing	P200 bias: OB > NW	In OB: Stroop RT bias ~ craving
							In NW: P200/P300 bias ~ craving
				P300 ERP			
				Stroop RT			
Phelan et al. [22]	15 long-term successful weight loss maintainers, 14 OB, 19 NW	Food-modified Stroop task	Words, referring to HC and LC foods	Stroop RT	Fasting state (>4 h)	Stroop RT interference HC words: successful dieters slower than OB/NW	–
Loeber et al. [26]	20 OB and 20 NW men and women	VPT	Pairs of pictures of foods and non-food items	VPT RT bias 50 ms	Restrain from eating 3 h prior to testing	–	–
Graham et al. [31]	15 OW/OB and 21 NW women	ET	Pairs of pictures of HC sweet foods; HC savory foods; LC foods	ET direction bias	–	Direction bias LC food: OW/OB > NW	–
				ET duration bias			

*BMI* body mass index; *ERP* event-related potentials; *ET* eye tracking; *HC* high-calorie; *LC* low-calorie; *NW* normal weight (BMI 18.5–25 kg/m^2^); *OB* obese (BMI > 30 kg/m^2^); *OW* overweight (BMI 25–30 kg/m^2^); *RT* reaction time; *VPT* visual probe task

However, as the results in Table 1 reveal, the approach-avoidance idea is not supported by all studies. Results have been inconsistent and seem to depend on methodological choices and the study design. There is a large variety in study designs, which makes it rather difficult to compare directly results of different studies. Moreover, one could raise questions on the validity of some of the used attention measures. In this review some issues are discussed, which could be taken into account when interpreting attention studies.

## Results Depend on Methodological Choices

In the studies summarized in Table 1 attention for food-related stimuli is derived from behavior in reaction time tasks, eye tracking, electrocortical activity (ERPs), or a combination of these measures. Among reaction time tasks the food-modified Stroop task and visual probe task were used, being classic attention tasks that have been applied in various other areas of attention research [7, 21]. In the food-modified Stroop task attention bias is derived from delayed reaction times in the color naming of food-related as compared to non-food-related words. Using a food-modified Stroop task, Nijs et al. [17] found no differences between weight groups with regard to the food-related interference effect. No differences between obese and “always” normal weight participants were found in the study by Phelan et al. [22] as well, although in this study slower reaction times to food words were found in a group of long-term weight loss maintainers with a history of overweight/obesity. These data suggest that an enhanced food-related Stroop interference might not be associated with overweight/obesity per se, but rather with attempts to restrict and control food intake. As discussed by Phelan et al. [22], it is unclear which index of information processing is measured with the food-related Stroop task. Delayed reaction times might reflect an emotional distraction by the content of food-related stimuli, because these are desired or perceived as threatening, or they could reflect an avoidance of food stimuli, because these produce a conflict between the desire to eat and the desire to maintain cognitive control. This might even differ between groups: for instance, Nijs et al. [17] found strong positive associations between Stroop interference scores and self-reported desire to eat in the obese group, but not in the normal weight group. There is also debate whether Stroop interference effects reflect a fast automatic or a slower strategic process [23–25].

In a food-modified visual probe task participants have to respond to a probe appearing at the position of one of two pictures (generally one food-related and one non-food-related), which were displayed simultaneously. Faster reactions occur when attention is already drawn to the position where the probe appears. With the visual probe task it should be possible to investigate the different processes of initial orientation and maintenance of attention, simply by adjusting the presentation duration of stimulus pairs (respectively, <200 ms and >200 ms [7]). However, some researchers question the usefulness of a visual probe task as an index of maintained attention. That is, when picture pairs are shown for a duration longer than 200 ms [14, 15, 16•], this gives participants the opportunity to shift their attention back and forth between the pair of stimuli. As reaction times are measured on just one specific point in time (ie, after the picture pair has disappeared), an “attention bias” could as well reflect the coincident direction of the eyes to one of the stimuli at that moment [7]. This makes it hard to interpret results from studies using the visual probe task with longer presentation durations, and it might explain why no weight group differences were found in maintained attention to food-related stimuli using such visual probe tasks [14, 15, 16•]. In general, the visual probe task is acknowledged to be a valid measure of attentional orientation when a short presentation time (<200 ms) of stimulus pairs is applied. With a stimulus presentation duration of 100 ms, Nijs et al. [16•] found evidence for an enhanced attention orientation to high-calorie food pictures in overweight/obese participants, but this was not corroborated by Loeber et al. [26], who displayed food-related picture pairs for 50 ms. Various other study design–related factors might underlie these inconsistent results, such as differences in the hunger/satiety state of participants of both studies.

The recording of electrocortical brain activity (electroencephalography/ERPs) during exposure to relevant stimuli is generally seen as a more direct measure of information processing. The amplitude of early (eg, P200) and later (eg, P300 and the following late positive potential, LPP) ERP components is thought to reflect the intensity by which cognitive/attentional resources are allocated to stimuli in early and later stages of information processing, and is modulated by the emotional relevance of stimuli [27, 28]. From addiction research, there is a general idea that particularly late ERPs are indices of selective and motivated attention, reflecting activity in the underlying reward system, as these have been found to be associated with subjective drug craving scores [8•]. In eating-related research it was also demonstrated that particularly late ERPs elicited by food-related stimuli are enlarged when (normal weight) participants are in a state of hunger (ie, in a state of enhanced motivation to eat) compared to a state of satiety [29]. However, in one of our studies it was demonstrated that the reverse pattern might be true in overweight/obese persons: P300 “bias” was strongly reduced, and even almost absent in overweight/obese participants who were food-deprived for more than 17 h compared to overweight/obese participants who were satiated [16•]. It was concluded that P300, as LPP [30], amplitude is sensitive to cognitive strategies, and the results were interpreted as an attempt to avoid attending to food stimuli in food-deprived overweight women. In a following study, we found differences between weight groups with regard to the P200 amplitude to food-related words [17], which is suggestive of an enhanced (more automatic) processing of food stimuli in overweight persons in early stages of information processing. The application of ERPs to study the processing of food-related information is still novel, and more research is necessary to study the modulation of ERPs by weight group and hunger state.

Probably the most direct measure of attention is the recording of eye movements during the exposure to two or more stimuli. Four studies employed eye tracking and similar attention parameters (ie, a gaze direction and duration bias as indices of, respectively, oriented and maintained attention). Castellanos et al. [14] found evidence for an enhanced direction and duration bias to low-calorie and high-calorie food pictures in obese participants relative to normal weight participants, but only after satiation. Nijs et al. [16•] found no differences in a (high-calorie) food-related direction and duration bias between weight groups and hunger/satiety conditions. Werthmann et al. [15] reported an enhanced direction bias (but no duration bias) to high-calorie food pictures in satiated overweight/obese persons relative to normal weight persons. Finally, Graham et al. [31] only found an enhanced direction bias to low-calorie (but not high-calorie) food stimuli in an overweight/obese compared to a normal weight group. These results demonstrate that not only the choice of attention task and parameters is an important factor, influencing study results, but also other differences in study designs might account for this. There were, for instance, differences across studies with regard to the gender of participants (males/females or only females, also see [32]), the food deprivation level of participants (ie, the degree of hunger/satiety), the satiation procedure (eg, milkshake vs normal meal), the general study design of hunger/satiety intervention studies (eg, within-subjects vs between-subjects design), the time of day when the experiment was conducted, the choice (eg, words vs pictures) and matching of food-related and control stimuli (Table 1). Some of these factors have also been found to influence findings on substance-related attention in addiction research [7, 8•].

Interestingly, studies using several methods for measuring attention underline the statement that different methodologies yield different results. For instance, Nijs et al. [16•] investigated correlations between attention-related outcomes of a visual probe task, eye tracking, and the P300 ERP component within one and the same study sample. No meaningful associations were found between the outcomes of various measures. This is surprising as these methodologies intended to measure similar underlying attention constructs. This lack of a clear relationship between various attention-related measures has been reported before in addiction [8•, 33], as well as eating-related literature [34]. Apparently, various attention measures tap different underlying mechanisms of information processing. Future studies should further try to clarify what is exactly measured with different instruments.

## Attention to Food Characteristic for All Overweight/Obese Persons?

In circa half of the studies differences in attention bias to food were investigated between overweight (body mass index [BMI] > 25 kg/m^2^) and normal weight participants, whereas in the other half obese participants (BMI > 30 25 kg/m^2^) were compared with normal weight persons. It is likely that addiction-like deficits in reward system functioning, and consequently an enhanced food cue-responsiveness, are particularly present in persons with a severe overeating/weight problem. For instance, one of the first neuroimaging studies, reporting on altered dopamine functioning in obesity, was conducted in participants with a BMI of 40 kg/m^2^ or more [12]. In the same vein, research in addicted persons suggests that the intensity of a substance-related attention bias is directly proportional to the quantity and the frequency of the substance use [7]. None of the mentioned studies has investigated differences between overweight and obese persons in food-related attention. Moreover, it might even be the case that enhanced attention bias to food is not characteristic for all obese persons, but rather for a subgroup of obese persons, for instance persons who have more serious overeating episodes with clear loss of control over eating, such as binge eaters. There is increasing consensus that binge eating disorder might be closer related to addiction than obesity per se [35–37]. In line with this point of view, Svaldi et al. [38] found, using an ERP methodology, that women with binge eating disorder demonstrate enhanced attentional engagement to specifically high-calorie food pictures compared to healthy overweight controls. Further, attention research in patients with eating disorders also suggests that enhanced attention bias to food is particularly seen in those patients characterized by binge eating [39]. In addition, obese bingeing women have also been found to report more food craving than obese non-bingeing women after being exposed to food stimuli [40]. For future attention studies, it might thus be recommended to focus on differences between obese bingeing, obese non-bingeing, overweight, and normal weight participants.

On the other hand, evidence suggests that enhanced attention bias to food is not exclusively seen in persons with overweight or an eating problem, but is also present in normal weight persons with an eating style that might render them vulnerable to become obese or develop an eating disorder in future. For instance, enhanced attention bias to food stimuli has been reported in external eaters [eg, 41, 42], in successful and unsuccessful dieters (eg, [18, 43]), and in chocolate cravers [19]. Interestingly, some researchers have started to investigate causal or directional relationships between enhanced food responsiveness and weight/eating problems. For instance, Calitri et al. [44] found that a cognitive bias to unhealthy food words in a food-related Stroop task predicted an increase in BMI over a 1-year period, whereas a cognitive bias to healthy food words was associated with a decrease in BMI.

## Associations Between Attention Bias and Subjective Craving/Hunger

In line with addiction research, several studies investigated associations between attention bias scores and subjective craving or hunger ratings. Whether positive associations are found again seems to depend on study design and methods, as well as weight group. For instance, Nijs et al. [16•] found positive correlations between ERP measures and subjective craving (as well as acute calorie intake) in normal weight participants, but not in overweight participants. This supports the idea that ERPs are an adequate index of food motivation in normal weight persons, but not in overweight. In overweight participants, however, positive correlations were found between behavioral outcomes of attention (ie, visual probe task, Stroop task) and craving, which was not seen in normal weight participants [16•, 17]. Castellanos et al. [14] reported general positive correlations between eye-tracking direction/duration bias and subjective hunger, whereas Werthmann et al. [15] only found a positive correlation between direction bias and subjective food craving, and this only in the overweight/obese group. This implies that some attention measures might be adequate indices of food motivation in one group of persons, but not in another group. One problem, perhaps explaining inconsistent correlational findings, might be the use of self-report questionnaires. It is well known that obese persons tend to under-report, to a greater extent than normal weight persons, eating- and weight-related issues, such as their body weight [45] and food intake [46]. Obese individuals might also tend to (purposively or not) under-report feelings of hunger or a desire to eat, perhaps because of social pressures or feelings of shame [47]. Researchers should always keep in mind that any self-report measure is unlikely to be a pure reflection of the subjective state it aims to measure [48], and that this might distort study results.

Interestingly, Werthmann et al. [15] and Nijs et al. [16•] added a bogus taste task to their attention study, to assess direct calorie intake of participants during the exposure to high-calorie foods. The assessment of direct food intake in this way might be a more straightforward measure of motivational food responsiveness than self-report questionnaires. In the study by Nijs et al. [16•], only in the hunger condition overweight/obese participants consumed significantly more calories than normal weight participants. This was interpreted as overweight/obese persons being particularly hyperresponsive to food in a state of hunger. However, Werthmann et al. [15] did find differences between weight groups after satiation, with overweight/obese persons eating more calories than normal weights. As mentioned earlier, differences in study findings might be the result of the satiation procedure or other methodological choices.

## Attention Bias as a Marker of Altered Reward Functioning

The incentive sensitization theory implies that the intensity of attention to reward-related stimuli is directly related to dopamine-based activity in the reward system. Although various studies have found evidence for altered reward functioning in obese persons compared to normal weight persons (eg, [12, 13, 49]), few studies have investigated whether attention bias scores are directly related to activity in reward-related brain regions. To our knowledge, only one study combined the measurement of food-related attention in a behavioral task with functional neuroimaging (functional MRI [fMRI]). Yokum et al. [50•] examined attentional bias in adolescent girls ranging from lean to obese using a food-modified attention network task. Outcomes of the behavioral task correlated positively with BMI, and were thus indicative for an enhanced orientation and reallocation of attention to food stimuli in persons with a higher BMI. Moreover, it was found that BMI correlated positively with activation in brain regions related to attention and particularly food reward, during initial orientation and reallocation of attention to appetizing food images. In addition, greater activation of the lateral orbitofrontal cortex (eg, involved in the evaluation of the reward value of a stimulus and decision making) during initial orientation to appetizing food cues predicted increase in BMI over a 1-year follow-up.

## Conclusions

In recent food-related attention bias studies, an interesting pattern of attention to food is seen in overweight/obese individuals, which is different from normal weight individuals: after an enhanced initial automatic orientation of attention to high-calorie stimuli (suggestive of an approach response), overweight/obese persons tend to show a more strategic attentional disengagement from these stimuli (suggestive of an avoidance response). However, as was discussed in this paper, study results are contradictory. This is possibly due to the use of different methodologies for measuring attention, which makes it difficult to directly compare study results. Therefore, it is too premature to draw straightforward conclusions on the question whether overweight/obese individuals demonstrate enhanced attention to food cues and to what degree this influences further food responsiveness, such as food craving and food intake. Although attention research is complex and challenging, it is of great importance to further investigate key issues, such as the identification of those (obese) persons in whom an attention bias to food is particularly present and problematic (eg, binge eaters), the exact circumstances in which an enhanced attention to food might be particularly present and problematic (eg, hunger/satiety or stress/emotions [51]), the directional/causal relationships between food-related attention bias and food intake/weight gain, and the underlying neurobiology of food-related attention. This knowledge may be important for developing or improving prevention or treatment programs (eg, based on attention retraining), which could support obesity-prone or obese individuals in dealing with the overwhelming exposure to food cues in daily life.
